# An introduction to standardized clinical nomenclature for dysmorphic features: the Elements of Morphology project

**DOI:** 10.1186/1741-7015-8-56

**Published:** 2010-10-04

**Authors:** Leslie G Biesecker

**Affiliations:** 1 Genetic Disease Research Branch, National Human Genome Research Institute, National Institutes of Health, Bethesda, MD, USA

## Abstract

Human structural malformations (anomalies or birth defects) have an enormous and complex range of manifestations and severity. The description of these findings can be challenging because the variation of many of the features is continuous and only some of them can be objectively assessed (that is, measured), among other factors. An international group of clinicians resolved to develop a set of terms that could be used to describe human structural malformations, under the general project name 'Elements of Morphology'. Here, the background to the project, progress to date, and the practical implementation of the terminology in research reporting is discussed.

## Background

Human structural malformations (anomalies, or birth defects) have a broad and complex range of manifestations and severities. The description of these findings can be challenging because the variation of the features is continuous and only some of them can be objectively assessed (that is, measured). Additionally, different specialties (embryologists, developmental biologists, surgeons, clinical geneticists, and so on) have developed descriptors for these findings that are based either on mechanistic, etiologic, or management considerations, and are thus laden with meaning, which can be invalidated by changing knowledge. Some terms have disparate definitions even within a specialty. Finally, a number of terms previously in common clinical use were disparaging (mongoloid slant, arachnodactyly, devil ear, and so on) and needed to be replaced with more neutral terms.

**Figure 1:**
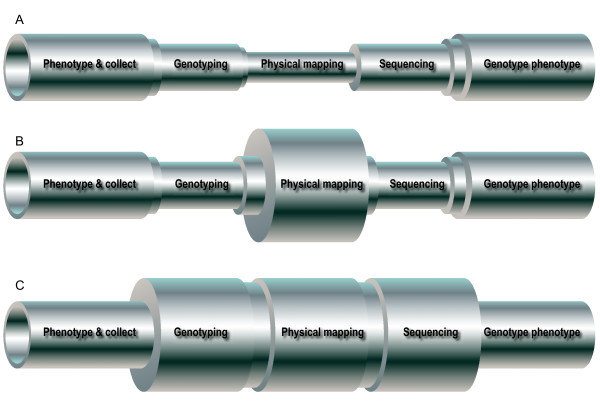
**A metaphorical pipeline that represents throughput of various processes involved in identifying the causative gene variation for a heritable disorder and its evolution**. **(a) **In the 1980 s and 1990 s, the throughput of genotyping and linkage mapping, physical mapping of genes, and sequencing of candidate genes were limiting factors in this overall process. **(b) **In the late 1990 s and into the first decade of the 21st century, the limitations of physical mapping were overcome by the Human Genome Project. **(c) **More recently, very high throughput chip-based SNP genotyping and next-generation sequencing technologies have eliminated the linkage and sequencing bottlenecks. Thus, the clinical analyses at the front and back ends of the process are now limiting and efforts to improve our ability to generate, archive, and analyze these data are necessary. Modified from with permission from [2].

The need to develop such a set of terms is driven by the ever-increasing throughput of biological methods, which are outstripping the ability of clinical analyses to properly phenotype patients for both research and clinical care. The processes of the elucidation of the etiology of these disorders can be represented as a pipeline of varying caliber, where the diameter of the pipe represents the throughput of the process. In the late 1980 s and early 1990 s, the limiting factors were entirely molecular: it was much more difficult and slow to genotype and map disorders, find genes within candidate regions and sequence them than it was to identify and clinically analyze the patients (Figure 1a). With the completion of the human genome project, physical mapping could be performed by interrogating a web browser (Figure 1b). Soon after, high throughput capillary sequencing and single nucleotide polymorphism (SNP) marker typing methods improved to lessen those impediments. Finally, with the advent of chip-based genotyping with > 10^6 ^features and next generation sequencing, molecular process throughputs are massive and no longer limiting (Figure 1c). Therefore, we have now reached a point where the ends of the process pipeline, which involves patient-related activities such as phenotyping and genotype-phenotype correlation, is the point limiting progress. The goal of implementing standardized terminology is therefore twofold: to increase the throughput of clinical analyses by developing a standardized common language that will allow development of databases for malformation phenotypes and to allow published case reports, case series, and genotype-phenotype correlation papers to serve as searchable repositories for clinical data. By harnessing these approaches, it is hoped that we can rapidly and effectively determine the etiology for hundreds or thousands of disorders and put these data to work, both as stimulants for basic science inquiries into mechanisms of normal and abnormal development and more directly for improved patient care.

## The Elements of Morphology project

**Figure 2:**
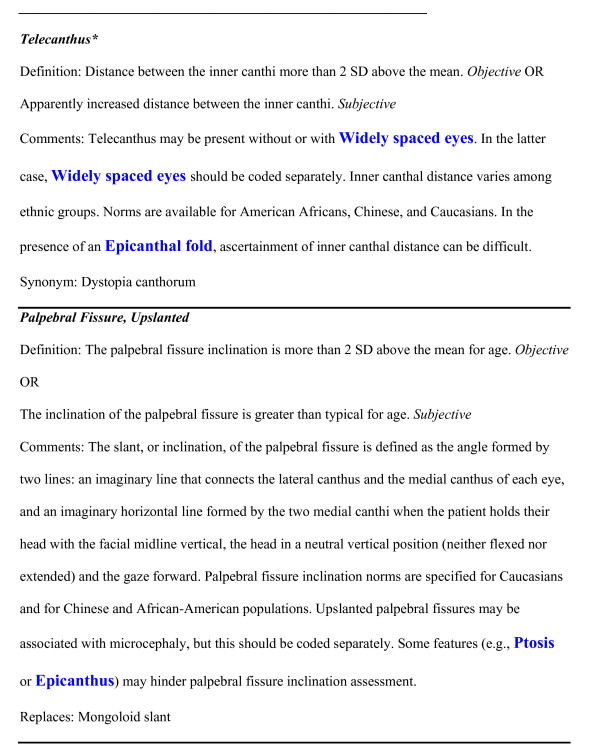
**Two illustrative examples of terms from the Elements of Morphology terminology set**. Note that these terms have been edited to remove indications of figures and citations, as those figures and references are not included in this commentary. In addition, the terms in blue are hypertext links in the terminology website but are not hypertext here as figures do not support hypertext.

The Elements of Morphology project was initiated to address the growing realization that the language used to describe human physical anomalies was inconsistent, incompletely defined, redundant, and in some cases confusing or even pejorative. On a practical level, these problems with the descriptive language are a barrier to text mining and database functionality [1]. To address this situation, an international group of clinicians resolved to develop a set of terms that could be used to describe human structural malformations. The attributes of this terminology set incorporated a number of important features [2]. Some of these features included; one-to-one correspondence of clinical terms to clinical manifestations, terms should be linked to appropriate, validated qualifiers, terms should not subsume multiple features if those features can occur alone, the terminologic set should be versioned so that it can be updated and referenced to prior versions. This effort was initiated in 2005 and resulted in a series of papers that described the rationale for the terms [3] and defined an initial list of terms in six categories (head and face [4], periorbital [5], nose [6], mouth and oral region [7], ear [8], and hands and feet [9]). Each of the terms was assigned a preferred descriptor, a one or two sentence definition, alternative objective and subjective definitions when appropriate, a list of acceptable synonyms, and a list of terms that are supplanted or replaced by the preferred term (see Figure 2 for examples). Definitions will be developed for another set of terms in the coming year. A web-based version of the terminology is also available http://elementsofmorphology.nih.gov/. This website includes the full text and the photographs of all of the definitions, an index of terms and synonyms and a hypertext linked anatomical figure to facilitate rapid location of relevant terms, and a feedback and commentary site for each term that allows users to post queries and suggest corrections, modifications, or even additional terms.

## Implementation of the standardized terminology

Standards of terminology and reporting have been suggested; however, without good uptake by researchers and editors, confusion over terminology will persist. It was the intent of the group that the guidelines and suggestions would be taken up by the wider research community. These terms would be very useful for phenotype databases. The human phenotype ontology system [10] has begun to incorporate the Elements of Morphology terms and definitions into their system. An example of a practical implementation of the terms is the genotype-phenotype databases for the International Standards for Cytogenomic Arrays consortium (http://iscaconsortium.org/ and see also [11]).

Going forward, it would be ideal for journal editors to determine that it is appropriate to ask authors of papers that describe malformations in humans to conform to this terminology. Efforts are underway to implement this terminology nomenclature for the *American Journal of Medical Genetics *and the *American Journal of Human Genetics*, in addition to the *BMC series*. The following approach is suggested for authors to follow, and editors to endorse for their publications:

1. For every term in case report descriptions, tables, and figure legends, authors should review the malformation terminology website or terminology papers (both cited above) to determine if the term is acceptable.

2. For terms that have alternative objective and subjective definitions, if the subjective definition is used, this should be specified. For example: 'The patient had telecanthus (subjective)'. If an objective definition is used for a quantitative finding, the measurement must be specified and in this case it is not necessary to specify that it is objective, since that is redundant. For example: 'The patient had telecanthus (inner canthal distance 3.9 cm, > 2 SD)'.

3. Manuscripts may include terms listed as 'synonyms' (see example 1, figure 2: ***Telecanthus ***re: the synonym dystopia canthorum), although this is not preferred.

4. Manuscripts may not include terms listed under 'replaces' (see example 2, figure 2; ***Palpebral fissure, upslanted***).

5. A hyperlink (where possible) for the first occurrence of each term in a report to the malformation terminology website, or a mirror site, to allow readers to readily access definitions and example photographs of the finding.

In support of this, the *BMC *journals have created wording about the terminology for their instructions for authors, strongly encouraging compliance with the recommended terminology from the elements of morphology project, and it is the hope that other journals will follow their lead in supporting this important issue. It is further hoped that this article provides some practical guidance on how to do so.

Authors will need to use judgment in specifying features within tables. It is recognized that specifying, for example, an objective finding such as telecanthus could be cumbersome. A recommendation is that the paper should include a concise table (for example see Table 1) that simply lists 'telecanthus' or an indicator such as a '+' or 'tick' symbol for the finding but this table would link out to a supplementary data sheet for each patient that lists the findings (for example see Table 2).

**Table 1:** A hypothetical clinical features table that would appear within the primary manuscript

	Macrocephaly	Widely spaced eyes	Telecanthus
Patient 1	+	+	+

Patient 2	-	+	NA

Patient 3	+	-	+

+ = feature present; - = feature absent; NA = feature not assessed.

**Table 2:** A hypothetical detailed clinical data table format that could be included as supplemental data with a manuscript; these data for each patient support and extend the data presented in the above summary table

	Patient 1	Patient 2
Sex	Male	Female

Age at examination	12 months	12 months

Weight	10.2 kg (approximately 50th centile)	9.8 kg (approximately 50th centile)

Length	75 cm (approximately 50th centile)	73 cm (approximately 50th centile

Head circumference	49.5 cm (> 97th centile)	48.5 cm (> 97th centile)

Interpupillary distance	5.5 cm (> 2 SD)	5.5 cm (> 2 SD)

Inner canthal distance	Increased (subjective)	1.8 cm (normal)

Note that these supplementary clinical data support all of the conclusions in Table 1 by providing raw data. The supplementary data also distinguish objective from subjective findings and Table 1 distinguishes the absence of data on a finding (by using the 'NA' indicator) from a normal finding.

## Conclusions

The display of data in standardized formats, using defined terms, traceable to example images, commentaries, and norms, distinguishing absent findings from those not assessed, and distinguishing subjective from objective assessments, will allow readers and data miners to independently assess underlying data and conclusions and use case reports and other published clinical analyses to make new discoveries in the future. Like all new approaches to publishing and displaying data, these standards and approaches will need to evolve, and the BMC journal community, and hopefully all biomedical journals, should participate in this process. All of the editors look forward to feedback from the readers and authors on ways to improve and refine our approach to this challenge.

## Competing interests

The author declares that he has no competing interests.

## Pre-publication history

The pre-publication history for this paper can be accessed here:

http://www.biomedcentral.com/1741-7015/8/56/prepub
